# Harnessing Multi-Photon Absorption to Produce Three-Dimensional Magnetic Structures at the Nanoscale

**DOI:** 10.3390/ma13030761

**Published:** 2020-02-07

**Authors:** Matthew Hunt, Mike Taverne, Joseph Askey, Andrew May, Arjen Van Den Berg, Ying-Lung Daniel Ho, John Rarity, Sam Ladak

**Affiliations:** 1 School of Physics and Astronomy, Cardiff University, Cardiff CF10 3AT, UK; HuntMO@cardiff.ac.uk (M.H.); AskeyJW@cardiff.ac.uk (J.A.); MayA1@cardiff.ac.uk (A.M.); VanDenBergA@cardiff.ac.uk (A.V.D.B.); 2 Department of Electrical Engineering, University of Bristol, Bristol BS8 1TH, UK; Mike.Taverne@bristol.ac.uk (M.T.); daniel.ho@northumbria.ac.uk (Y.-L.D.H.); John.Rarity@bristol.ac.uk (J.R.); 3 Department of Mathematics, Physics and Electrical Engineering, Northumbria University, Newcastle NE1 8ST, UK

**Keywords:** two-photon lithography, magnetism, nanoscale, three-dimensional, nanostructures

## Abstract

Three-dimensional nanostructured magnetic materials have recently been the topic of intense interest since they provide access to a host of new physical phenomena. Examples include new spin textures that exhibit topological protection, magnetochiral effects and novel ultrafast magnetic phenomena such as the spin-Cherenkov effect. Two-photon lithography is a powerful methodology that is capable of realising 3D polymer nanostructures on the scale of 100 nm. Combining this with postprocessing and deposition methodologies allows 3D magnetic nanostructures of arbitrary geometry to be produced. In this article, the physics of two-photon lithography is first detailed, before reviewing the studies to date that have exploited this fabrication route. The article then moves on to consider how non-linear optical techniques and post-processing solutions can be used to realise structures with a feature size below 100 nm, before comparing two-photon lithography with other direct write methodologies and providing a discussion on future developments.

## 1. Introduction

Optical lithography was first successfully utilised in the context of modern microelectronics in 1955, when Andrus and Bond who both worked at Bell Labs adapted photoengraving techniques in order to define micron-scale windows through which impurities could diffuse [1]. The technique provided a controlled means to produce the necessary n- and p-type regions within semiconducting devices. Just two years later in 1957, Jay Lathrop and James Nall, both working at the U.S. Army’s Diamond Ordnance Fuse Laboratories, successfully patterned metallic strips with a width of 200 μm as interconnects between Ge-based transistors [2]. Lathrop and Nall used the term “photolithography” to describe their process. These events effectively mark the birth of optical lithography which has driven a technological revolution in electronics.

It was not until the 1960s onwards before photolithography was commonly used to pattern magnetic materials. Here, it was used to understand the underlying domain structure of thin-film elements (usually with sizes of order 10 microns) and to test relatively new continuum-based theories, but also to realise magnetic bubble memories, first envisioned by Bell Labs scientists [3]. Though such bubble memories were later commercialised, they were not competitive against the hard disk drive and the emerging random access memory (RAM) technologies.

In 1979, photolithography was first used commercially in magnetic hard disk drive data storage devices. This marked the dawn of a new era for magnetism. By patterning thin-film read heads and utilising the anisotropic magnetoresistance effect, a significant increase in storage density was possible over previous generation systems relying upon inductive sensors [4]. Increasing demands for higher areal density later led to patterned, giant magnetoresistance (GMR) [5] and tunnelling magnetoresistance (TMR) [6] sensors, all relying upon more demanding photolithography processes. The most modern hard disk drives utilise high-anisotropy perpendicular media with either heat-assisted or microwave assisted magnetic recording, both utilising read-heads on a scale of order 50 nm. Thus, the advancement of the magnetic data storage industry, much like the transistor industry, has closely followed improvements in photolithography. Today, both industries utilise extreme ultraviolet lithography to define sub−50 nm feature sizes. 

Advances in optical lithography, as well as greater access to higher resolution electron-beam lithography techniques, provided academics with a means to study a wide range of patterned 2D magnetic microstructures from the 1980s onwards, with initial focus being industry-driven and exploring elements for use as higher density sensors in data storage [7]. Further studies explored more complex geometries and exploited the dependence of the demagnetisation energy upon nanostructure shape, to realise a plethora of spin textures including magnetic vortex states [8], single domain magnetic nanowires [9] and elements [10]. Access to patterned magnetic elements also provided access to new physics with strong potential for technological applications. Examples include the spin-torque effect [11,12] and fast domain wall motion in planar nanowires [13]. 

It was in 2004 that Stuart Parkin obtained the first patent upon magnetic racetrack memory, a data storage system based upon domain wall movement through three-dimensional magnetic nanowires [14]. Though initial demonstrations and prototypes were two-dimensional in nature, a move to the third dimension had clear advantages in terms of storage density. However, even before this pivotal point, interest was starting to increase in 3D nanostructured magnetic systems. For example, a large number of studies from the 1990s onwards investigated the use of electrodeposition into anodised alumina or ion-track etched templates in order to realise arrays of single material or multi-segmented cylindrical magnetic nanowires [15,16,17,18]. Alternative techniques were also being used to produce 3D magnetic nanostructures. For example, in 2002, chemical synthesis was used to fabricate spherical CoPt_3_ particles (particle size: 1.5–7.5 nm) which could self-assemble into complex 3D colloidal crystals [19]. In the same year, 3D cobalt nanocrystals were produced by application of a ferrofluid to oriented pyrolytic graphite and applying a magnetic field [20]. Such methods were innovative and provided a first taste of the interesting properties that may arise when nanostructuring magnets in three-dimensions. Perhaps what was lacking was a controlled means to produce nanomagnets of arbitrary 3D geometry. 

Meanwhile in the adjacent field of photonics, a new methodology known as two-photon lithography (TPL) was being exploited to produce complex 3D nanostructures [21]. The technique, which made use of pulsed lasers within the picosecond or femtosecond regime, was capable of defining any 3D geometry within a polymer, at a resolution of order 100 nm. From the 2000s onwards it has been used to great effect in order to realise photonic crystals [22,23,24,25,26], microfluidic channels [27] and cell scaffolds [28,29,30,31]. It would be another 15 years before the potential of two-photon lithography was exploited to realise 3D magnetic nanostructures.

Today, 3D Nanomagnetism has come of age [32], providing the potential to host a plethora of new physics including spin textures locked by topological protection [33], ultrafast domain wall motion [34], controlled spin-wave emission [35,36], magnetochiral effects [32] and curvature-driven energies that produce new effective interactions [37,38,39,40]. A thorough investigation of these phenomena now demand new fabrication techniques that enable controlled sub-100 nm growth of magnetic materials in three-dimensions.

In this article, we will review the recent use of two-photon lithography and associated processing in order to realise a wide range of 3D magnetic nanostructures including nanoelements, nanowires and 3D nanolattices. In Section 2, we will outline the basic physics of two-photon lithography and outline expressions for minimum feature size in terms of experimental quantities. In Section 3, we will detail the different means to cast a polymer into magnetic materials and look at recent examples in the literature. In Section 4, the means by which the feature size and resolution can be reduced will be explored, again surveying recent examples. In the final section, the current state of the art with respect to TPL will be assessed and compared to other alternative fabrication strategies.

## 2. Experimental Setup and Physics of Two-Photon Lithography

Standard photolithography, widely used in the fabrication of two-dimensional structures and devices, generally relies on single-photon absorption to trigger a chemical reaction in a medium known as a photoresist [41]. Such resists can be referred to as ‘positive tone’ photoresists where the reaction serves to depolymerise an already hardened resist, or ‘negative tone’ where the reaction is used to cross-link monomers into a stable structure [42]. In a standard 2D lithography process, areas of resist are masked off and the open regions flooded with light to selectively expose the resist according to the intended design. The device is then submerged in a developer solution (in effect a solvent of the resist) to remove the exposed resist for the case of positive tone, or the unexposed resist for the case of negative tone. Two-photon lithography, the physics of which are detailed below, can be used with either type of resist. However, our general discussion in this section refers to the fabrication of structures from negative tone resist.

A negative photoresist generally consists of the three following types of chemicals: a monomer, which is the substance to be cross-linked and of which the final fabricated structure will consist; a photoinitiator, the substance that triggers the polymerisation process after absorbing a photon; and an optional inhibitor, a substance that helps terminate the polymerisation process. The monomer is transparent to the wavelength of the light used in the photolithography process (typically UV), while the photoinitiator absorbs it. Under illumination, the exposed resist undergoes a photopolymerisation process which occurs in four steps. Firstly, when a photoinitiator molecule absorbs a photon, it decomposes into a number of its underlying constituents and in the process produces free radicals [43,44]. These free radicals chemically react with a monomer molecule, forming a radicalised monomer. The radicalised monomer can react with and join to other monomer units, creating a growing polymer chain. The polymerisation process eventually terminates in one of the following ways: combination of the radicalised monomer with other active radicals, combination with other radicalised monomers, reaction with inhibitors or when no monomers are left.

For negative tone photoresists, in order to obtain a solid structure after development, a sufficient amount of monomer needs to be polymerised, so that it does not get dissolved in the solvent. While an exact quantification of the amount of monomer that gets polymerised on exposure is difficult, a simple model that can be applied is the threshold model. If the local exposure dose *D* is higher than a certain threshold dose *D_th_*, the exposed area is polymerised. The exposure dose is approximately proportional to the generated free radicals and therefore to the excited photoinitiator molecules and thus to the number of absorbed photons. In order to write arbitrary structures, it is necessary to only expose desired areas. This is trivial to do for 2D fabrication by using a simple optical mask. For more complex 3D geometries, it becomes necessary to focus the light using a high numerical aperture (NA) lens, so that only a limited volume (‘voxel’) of the photoresist around the focal point is exposed with enough intensity to reach the threshold dose. 

In the case of single-photon lithography, the light entering the photoresist is attenuated by single photon absorption. According to the Beer-Lambert law [45], the absorbance *A* of the photoresist increases in proportion to its thickness *z* and concentration or density *c*:

(1) A(z)= εcz

where *ε* is absorptivity (m^2^∙mol^−1^), *c* is the concentration of photoinitiator (mol∙m^−3^) and *z* is the depth into the photoresist (m). The absorbance *A*(*z*) is related to the transmittance *T*(*z*) (ratio of transmitted intensity *I*(*z*) to incident intensity *I*_0_) by:

(2) $$T\left(z\right)=\;\frac{I\left(z\right)}{I_{0}}=\;10^{-A\left(z\right)}$$

The transmitted intensity therefore decreases exponentially within the photoresist:

(3) $$I\left(z\right)=\;I_{0}e^{-\ln\left(10\right)\varepsilon cz}$$

(4) $$\frac{dI}{dz}=\;-\ln\left(10\right)\varepsilon cI\left(z\right)$$

**Figure 1:**
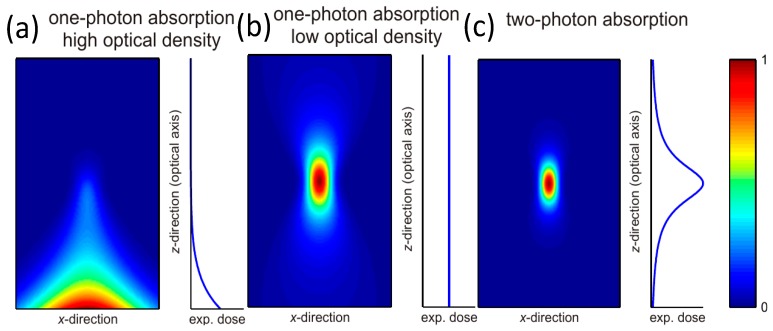
Illustration of laser intensity profile for (**a**) resist with high-concentration photoinitiator; (**b**) resist with low-concentration photoinitiator; (**c**) two-photon absorption profile [44].

Figure 1a shows the light intensity profile of a focused Gaussian beam in a photoresist with a high concentration of photoinitiators (high optical density). In that case, the beam is strongly attenuated and cannot reach deeper into the photoresist, preventing the creation of 3D structures. Figure 1b shows the light intensity profile of a focused Gaussian beam in a photoresist with a low concentration of photoinitiators (low optical density). Now the beam intensity is strongest at the focal point. To write a plane at a specific depth in the photoresist, all that is necessary is to focus the light at the specific depth and then raster scan the *x-y* plane. Unfortunately, the exposure dose is also additive in the sense that the dose *D’* after *n* exposures of dose *D* is *n* × *D*. However, when one integrates the intensity in each *x-y* plane, one finds that the intensity is almost constant along *z*. This means that if the beam raster scans a given *x-y* plane, the final exposure dose is the same for all points, independently of *z*, leading to the creation of a solid block of polymerised material instead of a plane as one would expect. 

The solution to this is to use two-photon polymerisation. Figure 1c shows an example of two-photon absorption (2PA) which is a non-linear optical process where molecules are excited from a ground state to an excited state via the absorption of two photons instead of one.

**Figure 2:**
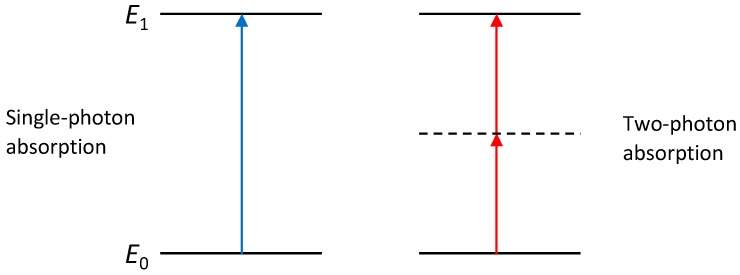
Energy level schematic for single photon absorption and two-photon absorption processes. Dotted line indicates imaginary intermediary state.

For photons to be absorbed, their total energy must be larger than the energy gap Δ*E* between the ground and excited states of the photoinitiator. This means that either one photon of frequency *ν* so that *hν* ≥ Δ*E* must be absorbed (one-photon absorption (1PA)) or two or more photons of frequency *ν_i_* so that Σ*hν_i_* ≥ Δ*E* (multi-photon absorption) (Figure 2). In the case of multi-photon absorption, the photons must arrive at the photoinitiator within a small enough time window. It is therefore less likely than 1PA. The probability of absorption (absorption rate) is proportional to *I^n^* where n is the number of absorbed photons (assuming photons of the same frequency *ν*) and *I* the intensity of the light at the given frequency *ν*. Two-photon absorption (2PA) therefore requires much stronger intensities than one-photon absorption to make the process efficient enough for lithography. Table 1 summarises the properties of 1PA and 2PA. 

Efficient multiphoton absorption can be readily obtained using femtosecond pulsed lasers. As can be seen in Figure 1c, due to the proportionality to *I*^2^ instead of *I*, the exposed area is much smaller. In addition, when integrating over the *x-y* plane, one finds a peak in the focal plane. This allows setting the power so that only the volume enclosed within the focal point (the voxel) receives sufficient exposure to be polymerised, thus enabling the creation of the desired 3D plane or any arbitrary 3D structure.

Two-photon lithography (TPL) has seen wide use in the rapid prototyping of structures on the mesoscale, in fields such as micro-optics, micro-fluidics and biological sciences. When considering its use for magnetic nanostructure fabrication the minimum feature size obtainable is of significant interest, particularly when trying to achieve single domain behaviour in the resultant structures. For negative tone photoresist Zhou et al. provide a comprehensive theoretical analysis of the voxel size based on the density of radicals and the above exposure mechanism [46], which culminates in the following expression for voxel diameter:

(5) $$d\left(P,t\right)=\;\omega_{0}{\left(\ln\frac{\sigma_{2}{I_{0}}^{2}n\tau }{C}\right)}^{\frac{1}{2}}$$

where *d* is the voxel diameter as a function of laser power *P* and *t* is the total processing-irradiation time, *n* = *ft* is the number of pulses, *f* is the repetition frequency of the laser, *ω*_0_ is the beam waist (minimum radius of beam at focus), *σ*_2_ is the effective two-photon radical absorption cross-section, *I*_0_ is the photon flux at the beam centre, *τ* is the laser pulse width and *C* is defined as the following equation:

(6) $$C=\ln\left[\frac{\rho_{0}}{\left(\rho_{0}-\rho_{th}\right)}\right]$$

where *ρ*_0_ is the density of free radicals at the focal plane and *ρ_th_* is the threshold density of free radicals.

And the axial length of the voxel is similarly described by:

(7) $$l\left(P,t\right)=2z_{R}{\left[{\left(\frac{\sigma_{2}{I_{0}}^{2}n\tau }{C}\right)}^{\frac{1}{2}}-1\right]}^{\frac{1}{2}}$$

where *z_R_* is the Rayleigh length, which is defined as the following equation:

(8) $$z_{R}=\;\frac{\pi {\omega_{0}}^{2}}{\lambda }$$

For positive tone photoresists, the resultant linewidth following TPL exposure can be derived by considering a reaction kinetics model as shown by Cao et al. which results in the following expression for voxel diameter [47].

(9) $$d=w\left(z\right)\sqrt{\ln\left[\frac{4C^{\prime }\eta^{2}P_{laser}^{2}t}{f\tau {\left(\pi h\nu w^{2}\left(z\right)\right)}^{2}ln\frac{M_{0}}{M_{th}}}\right]}$$

where *z* = distance from laser focus, *w*(*z*) = beam radius at position *z*, *η* = transmittance of the objective lens, *P_laser_* = incident laser power, *t* = processing time, *f* = repetition frequency of the laser, *τ* = pulse width, *ν* = frequency of light, *M*_0_ = initial concentration of photoinitiator in the ground state, *M_th_* = threshold amount of dissolvable photoinitiator. The factor *C’* relates the quantum efficiency (Φ), two-photon absorption cross-section (*δ*), the Einstein coefficient of absorption (*A_E_*) and a rate constant (*C*_0_) as shown below:

(10) $$C^{\prime }=\;\frac{\Phi \delta C_{0}}{A_{E}+C_{0}}$$

While TPL is a powerful technique for realising polymer-based structures at the nanoscale, the creation of magnetic nanostructures requires further processing steps as detailed below.

**Table 1:** Comparison of optical properties for single photon absorption (1PA) versus two-photon absorption (2PA).

Optical Property	1PA	2PA
transmitted intensity, *I* [48]	$I\left(z\right)=\;I_{0}e^{-\ln\left(10\right)\varepsilon cz\left(z\right)}$	$I\left(z\right)=\;\frac{I_{0}}{1+\beta zI_{0}}$
$\frac{dI\left(z\right)}{dz}$ [49]	$\frac{dI\left(z\right)}{dz}=\;-\ln\left(10\right)\varepsilon c\times I\left(z\right)$	$\frac{dI\left(z\right)}{dz}=\left(-\beta \right)\times I{\left(z\right)}^{2}$
transmittance [50] $T\left(z\right)=\;\frac{I\left(z\right)}{I_{0}}=\;10^{-A\left(z\right)}$	$T\left(z\right)=\;e^{-\ln\left(10\right)\varepsilon cz\left(z\right)}$	$T\left(z\right)=\;\frac{1}{1+\beta zI_{0}}$
decadic absorbance [51,52] $A\left(z\right)=\;\frac{-\ln\left(T\right)}{\ln\left(10\right)}$	A(z)= εcz	$A\left(z\right)=\;\frac{\ln\left(1+\beta zI_{0}\right)}{\ln\left(10\right)}$ $≅\;\frac{\beta z}{\ln\left(10\right)}\times I_{0}$
absorption probability/rate [44,50] $P\;\propto \;I^{n}$	P ∝ I	$P\;\propto \;I^{2}$

## 3. Fabrication of Magnetic Nanostructures with Two-Photon Lithography

### 3.1. TPL and Electrodeposition

Electrochemical deposition (or electroplating) is a technique for coating a conductive surface/structure with a desired metal, via the reduction of the corresponding ionic metal salt dissolved in aqueous solution, governed by an externally applied current controlled by a power supply. This system is referred to as an electrochemical cell, with the simplest example consisting of a cathode (negatively charged, upon which reduction of the desired metal occurs), and an anode (positively charged, upon which oxidation occurs).

This technique presents a powerful means for 3D nano-fabrication, especially of magnetic nanostructures, and has been used extensively in various forms to fabricate magnetic nanowires [33,53,54], multi-segmented nanowires [55], nanotubes [56], and for the coating of complex 3D structures [57]. Electroplating of nanowires and nanotubes is often achieved using nanoporous anodised aluminium oxide (AAO) templates, where a conductive layer at the end of the pores acts as the cathode upon which the nanowires grow. These templates have been used to fabricate FeNi nanowires [33,53] which have been used to study domain wall nucleation and Bloch-point dynamics; and also been used to grow multi-segmented alternating magnetic/non-magnetic nanowire arrays FeCo/Cu [55] and Co/Au [58] for magnetisation ratchet and domain wall pinning studies, respectively. However, these templates offer limited geometry dictated by the membrane pores, so one cannot tailor the geometry of the wires. Alternatively, one can fabricate a porous template through ion track etching [59,60], where a porous polycarbonate foil is bombarded with high energy ions and then these tracks are selectively etched in a strong base. However, the drawback with this method is again limited control over the geometry and pore surface roughness.

The biggest attraction of using TPL to fabricate templates for electrochemical deposition is its ability to generate templates where the pores are of truly arbitrary 3D design. 

**Figure 3:**
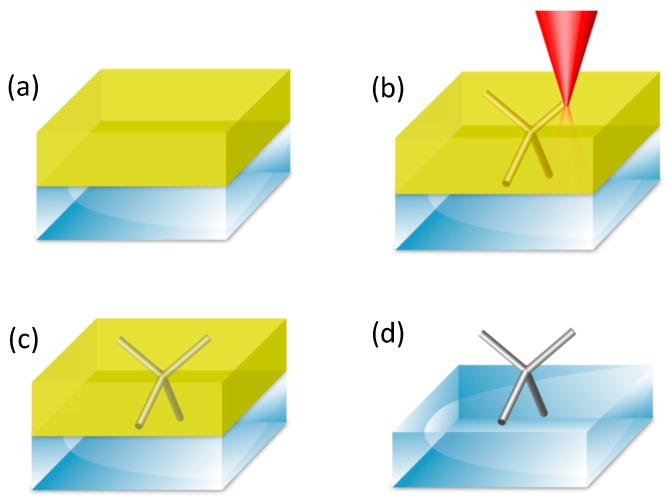
Use of TPL and electrochemical deposition to fabricate 3D magnetic nanostructures. (**a**) Spin-coating of a positive resist onto a conductive substrate; (**b**) Two-photon lithography of a 3D structure into the positive resist; (**c**) Electrodeposition of magnetic material into the channels; (**d**) Lift off of the resist.

The technique can be employed with both positive and negative tone photoresists, each with advantages and disadvantages. Using TPL with positive tone photoresist is relatively straightforward: after spin-coating a conductive though transparent substrate with a uniform layer of photoresist, the desired 3D geometry is TPL exposed with the caveat that the design must span the entire thickness of the resist layer (or include an entry channel for the electrolyte bath). After development, this results in a set of open channels in the resist layer which function as a template for electrodeposition [61] (Figure 3). 

The methodology described here exploits an attractive advantage of electrodeposition—the fabrication of arbitrary solid 3D magnetic geometries. Unfortunately, it is extremely challenging to reach the theoretical minimum feature size for TPL with positive tone photoresists. During the development process, dark erosion, where unexposed photoresist is also removed at a rate dependant on the development time [62], serves to widen the template pores which places a lower limit on the available feature size, which after electrodeposition yield multidomain systems. 

One can instead drop-cast a negative tone photoresist to produce solid 3D polymer geometries which can act as a tailor-designed nanoporous template, similar to that of an AAO template, that can then be filled using electrodeposition. This method offers a much smaller achievable feature size for the magnetic geometries as negative tone photoresists do not suffer from significant dark erosion effects. However, a significant issue with this approach is that the highly cross-linked polymer is incredibly resistant to removal via chemical means (in stark contrast to the positive tone photoresist route), and typically requires the use of reactive-ion plasma etching in order to dissociate the cross-linked molecules. Additionally, since one has to expose the inverse template volume, such templates typically occupy a small surface area of the substrate requiring patterned electrodes as opposed to a uniform coating as used for positive resist. 

The approach was first used with a positive resist for 3D magnetic nanostructure fabrication. Here, microhelix swimmer robots with magnetic components were fabricated [63,64]. Zeeshan et al. used a hybrid fabrication approach, using TPL written templates in positive tone photoresist before electrodepositing a CoNi block at the bottom of the template before filling the remaining helix with poly(pyrrole) polymer. The resist surrounding the microdevices was then removed with acetone and the devices detached from the substrate using a micromanipulator. The microrobots formed with this technique had a 650 nm wide and 1.6 µm tall cross section for the helical tail and measured 20 µm in total length. Alcântara et al. also used a positive photoresist but instead designed purely helical templates with TPL and filled the template fully with Fe. The feature sizes in this study were at the mesoscale, with the helix cross-section on the order of micrometres and the total length approximately 100 µm. Both studies were able to demonstrate controllable motion of the devices under applied external magnetic fields.

**Figure 4:**
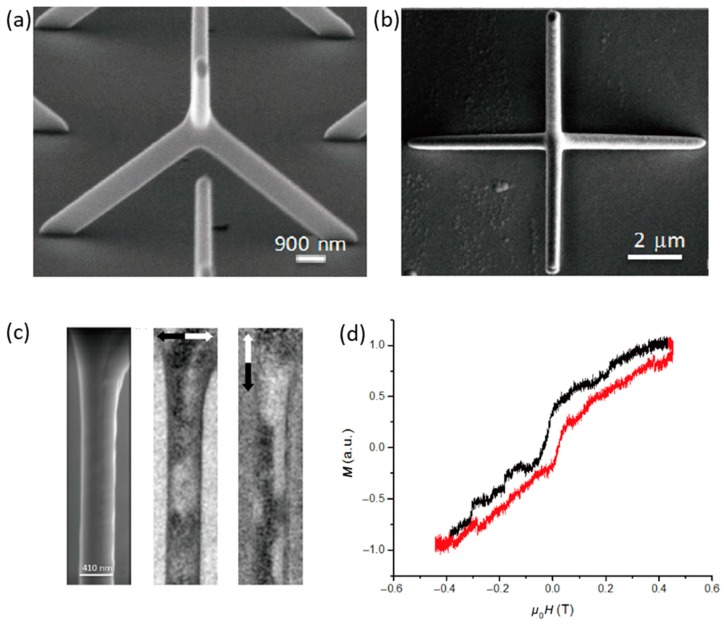
(**a**) Tilted SEM image of a single Co tetrapod structure; (**b**) Top-down SEM of a single Co tetrapod structure; (**c**) SEM micrograph of an individual wire within a tetrapod structure (left) and spin-polarised SEM images showing x and y-components of magnetisation in an as-deposited sample (middle and right); (**d**) Longitudinal MOKE loop obtained from tetrapod array with field applied along the projection of the lower wires onto the substrate [65].

The methodology has also been utilised to realise smaller 3D structures with complex magnetic interactions, such as cobalt tetrapods [65] with a smallest achieved wire width of 430 nm (Figure 4a,b). These cobalt tetrapods were investigated using spin-polarised scanning electron microscopy (spin-SEM) to probe the magnetisation at the 3D vertex (Figure 4c), and magneto-optical Kerr effect magnetometry (MOKE) identified the existence of plateaus in the switching field arising from the unique geometry (Figure 4d). Such structures were then further probed with time-resolved MOKE [66]. Here an optically pumped setup was used to measure the spin-wave modes at the junction of the tetrapod, which by comparison to micro-magnetic simulations could be visualised. 

The negative tone resist approach has also been demonstrated by Schürch et al. to fabricate 3D Ni microbridge structures [67]. The study included simulation of the electrodeposition process since 3D templates introduce additional complexities such as varying current densities and bottlenecks in both lateral and vertical directions. The simulations aided the fabrication process and the group was able to fabricate fully solid structures with a minimum feature size of 6 µm that were left freestanding on the substrate after removal of the template via oxygen plasma. 

An exciting development in this area is the creation of conductive scaffold templates for electrodeposition purposes [57]. Gliga et al. recently demonstrated proof of principle for the technique by fabricating a buckyball scaffold using TPL, applying etching post processing to reduce the feature size (see Section 4) before coating the polymer surface with a layer of iridium using atomic layer deposition. The conductive buckyball was then made magnetic by electroplating a layer of Ni approximately 30 nm thick onto the underlying 300 nm diameter wires, forming a connected lattice of nanotubes. Thus far, no magnetic characterisation has been performed on such structures.

Electroless plating is a similar method to electroplating but without the necessity for an external bias due to the autocatalytic nature of the reaction. This process can in principle coat any surface (i.e., non-conductive) conformally. This has been used for the fabrication of magnetic nanotubes such as Ni-Fe-B [68] into AAO templates, where the coercivity was found to vary as a function of the concentration of Fe in the bath. Electroless plating has also been used to conformally coat PMMA nanospheres with various alloys [69], including Ni-P, Co-Ni-P, and Ni-Fe-P, where the thickness of the deposits can be precisely controlled through the immersion time in the electroless plating bath. Furthermore, Ag has been electrolessly deposited onto arrays of nanostructures fabricated through TPL [70], whereby the SU-8 photoresist was modified using an RF plasma in order to facilitate the growth of the silver particles. Additionally, Ni-P electroless plating and TPL has been combined in order to realise magnetic micromachines that were remotely manipulated using external magnetic fields [71]. These structures were fabricated by first synthesising a bespoke composite polymer film, which was sensitised and catalysed, before using TPL to create the desired 3D geometry. This was then subsequently coated via electroless plating. 

### 3.2. TPL and Line of Sight Deposition

Line of sight (LOS) deposition techniques (such as thermal evaporation or sputtering) may be combined with two-photon lithography to rapidly realise complex 3D magnetic nanostructures. While this technique has largely been used for biocompatible devices [72,73,74], prior work has also demonstrated how these techniques may be combined for the fabrication of magnetic nanowires in complex 3D arrangements [75,76]. 

**Figure 5:**
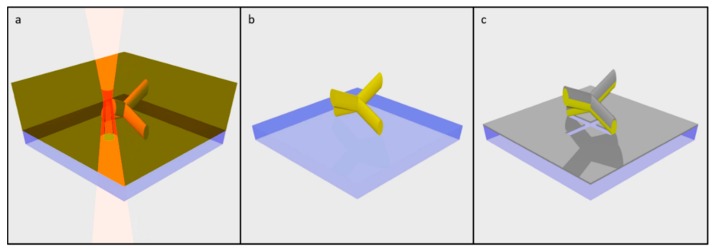
Illustration depicting the fabrication of a 3D arrangement of magnetic nanowires, via TPL and LOS deposition. (**a**) Exposure of photoresist during TPL; (**b**) Polymer scaffold after development of unexposed photoresist; (**c**) Resulting magnetic nanowires and sheet film, following deposition of a thin magnetic film.

A non-magnetic structure created through TPL using negative tone photoresist serves as a scaffold (Figure 5a,b) upon which a thin layer of magnetic material is deposited using either sputtering or thermal evaporation (Figure 5c). During the process, magnetic material is deposited onto the upper surface of the scaffold. Any surface directly below this is shadowed and remains uncoated during LOS deposition as illustrated in Figure 5c.

This simple yet versatile procedure enables the fabrication of complex 3D geometries and LOS deposition processes intrinsically yield high purity films. However, magnetic material is also deposited upon the substrate which may result in undesirable interactions. Carefully considered scaffold design can minimise these interactions by ensuring sufficient separation between the structures and the substrate. This allows local probe microscopy to determine the magnetic properties of the structure. However, the signal from the substrate will often be detected when performing optical or bulk magnetometry and therefore relevant subtraction techniques must be used. 

**Figure 6:**
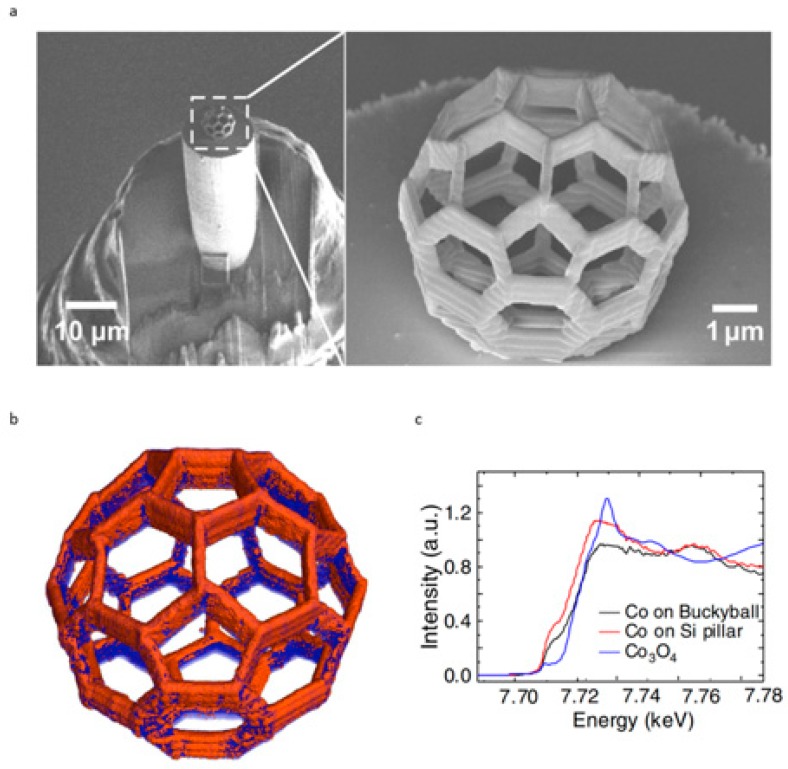
Physical characterisation of a cobalt-coated buckyball. (**a**) SEM images displaying the buckyball mounted upon a tomography pin (left) and a magnified image of the fabricated structure (right); (**b**) 3D rendering of the buckyball composition, obtained by x-ray tomography, cobalt is indicated by orange contrast whilst photoresist is blue; (**c**) Fluorescence spectra of cobalt deposited upon the polymer scaffold, upon the pillar and cobalt oxide detected in transmission [76].

In the first documented example of this technique, Donnelly et al. [76] produced a cobalt-coated buckyball with a nanowire feature size of 240 nm, directly written upon a 10 µm Silicon pillar (Figure 6a) demonstrating the substantial flexibility and precision of TPL. As a result, the fabricated structure could be transferred to a pin for x-ray ptychographic tomography imaging, allowing elemental characterisation with a spatial resolution of 25 nm (Figure 6b). By quantifying the electron density within the 3D structure, it was determined that the Co layer was oxidised, and this was further confirmed by micro-fluorescence experiments (Figure 6c).

**Figure 7:**
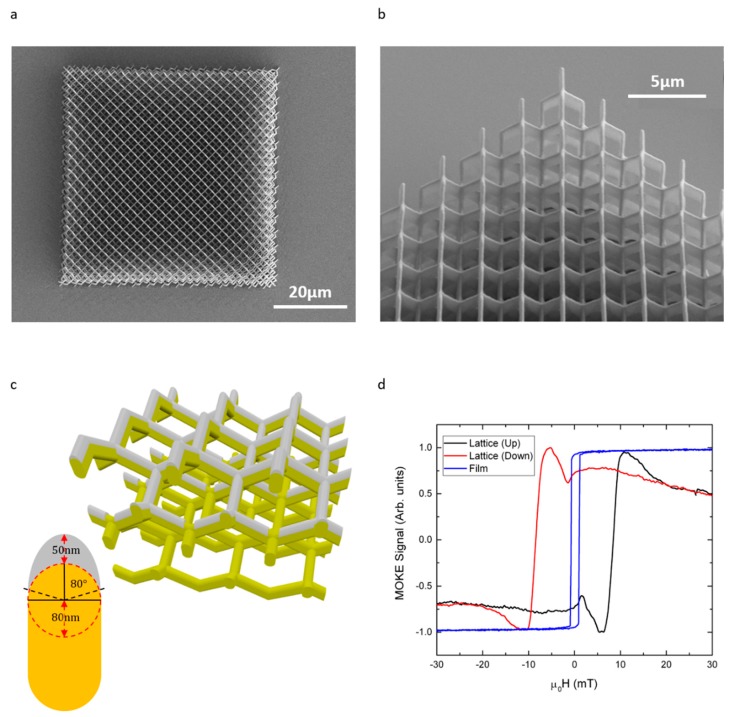
Structural and magnetic characterisation of a 3D N_i81_Fe_19_ nanowire lattice. (**a,b**) SEM images of the nanowire lattice observed from top view and a 45° tilt respectively; (**c**) Schematic of the Ni_81_Fe_19_ nanowires (grey) upon a polymer scaffold (yellow), where the effects of shadowing by upper nanowire layers during LOS deposition is evident. Inset: Nanowire cross-sectional geometry; (**d**) MOKE data captured from the sheet film (blue) and nanowire lattice—this is separated into up-sweep (black) and down-sweep (red) [75].

Recently, May et al. [75] realised a diamond lattice geometry comprised of high purity, single domain Ni_81_Fe_19_ (permalloy) nanowires with a novel curved cross-section, at a resolution of ~200 nm (Figure 7a–c). With LOS deposition, the permalloy was deposited upon the uppermost unit cell without depositing magnetic material on the lower scaffold layers, thereby minimising dipolar interactions between the magnetic nanowires and the sheet film upon the substrate. Additionally, the buried polymer layers act as optical scattering centres, allowing MOKE measurements to be performed with minimal contribution from the sheet film (Figure 7d). This study reports a first example showing that TPL and LOS deposition can rapidly yield extended arrays of single-domain magnetic nanowires in complex 3D geometries. Standard measurement techniques such as MOKE and magnetic force microscopy (MFM) were used to probe the magnetic properties, providing an exciting new path of investigation for 3D magnetic nanowires and artificial frustrated systems. Utilisation of other imaging techniques such as scanning transmission x-ray microscopy (STXM) [77] and high resolution Hall sensing [78] is expected to yield even more fruitful results.

The line of sight deposition technique has also been used to selectively coat parts of complex mechanical swimmer devices on the mesoscale [79]. Liao et al. fabricated a swimming device with a hinged flap using TPL and additionally patterned a sacrificial shell to cover the head of the device so that only the tail would be coated with a 50 nm layer of Ni and hence respond to an external field.

Iron has also been deposited onto TPL scaffolds comprised of wires with lateral feature sizes between 750 nm and 1 µm, using pulsed laser deposition [80]. To test the mobility of coated structures, Spanos et al. also fabricated mesoscale ‘lockyball’ structures coated with Fe and observed their movement in response to an external field when suspended in water.

## 4. Methods to Reduce Feature Size

In general, a reduction in feature size can either be obtained by changing the experimental apparatus, modifying the chemistry of the resist or by applying post-processing solutions.

Typically, the minimum lateral feature size of the voxel for a commercial TPL system operating at 780 nm wavelength is of the order of 200 nm, with the azimuthal feature size being up to 3–4 times larger. However, since the voxel is confined to a region within the focal point, by decreasing the wavelength it is possible to reduce the voxel’s size. An estimate of the voxel radius *r_xy_* for NA values > 0.7 has been shown to be [81]:

(11) $$r_{xy}=\;\frac{0.32\lambda }{\sqrt{2}\;{\left(NA\right)}^{0.91}}$$

**Figure 8:**
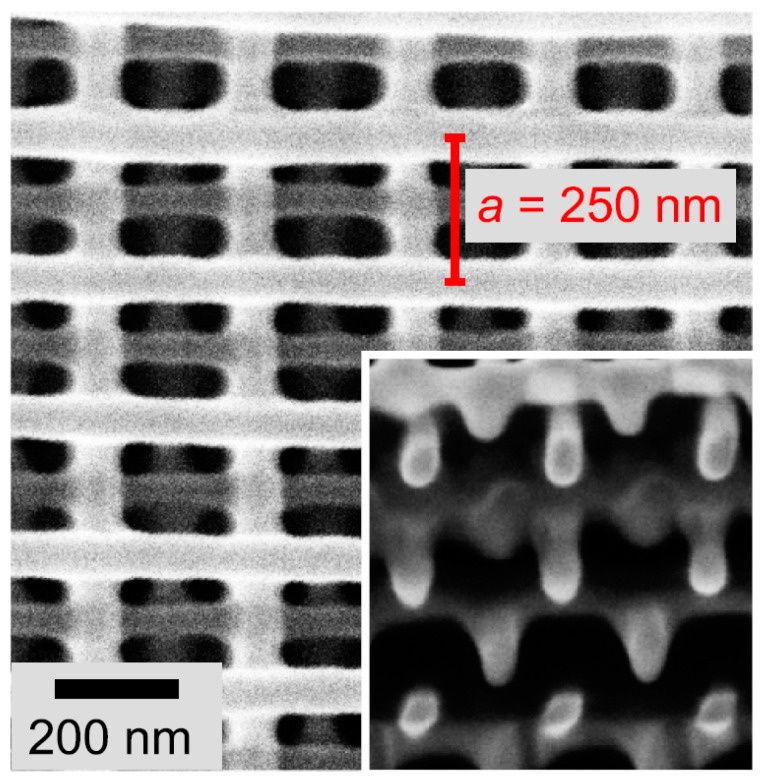
SEM image of a woodpile structure fabricated by TPL with a laser of wavelength 405 nm. Smallest line width is reported to be 68 nm. Reprinted with permission from ref [83] © The Optical Society.

This shows that the voxel diameter is directly proportional to the wavelength of the incident light [82]. Mueller et al. [83] developed a TPL setup using a 405 nm wavelength laser and achieved line width feature sizes of 68 nm (Figure 8). When switching wavelengths, however, a new resist where polymerisation is tuned to the new wavelength must normally be used. Here, the simple use of a common two-photon resist, with no photoinitiator provided sufficient non-linearity for 3D nanostructure fabrication [83]. Alternatively, chemicals can be added to the resist to reduce the voxel size by lowering the concentration of radicals available within the focal region. Park et al. [84] used the radical quencher 2,6-di-*tert*-butyl-4-methylphenol (DBMP) with the resist SCR500 to reach a lateral feature size of approximately 100 nm but noted that the strength of the resultant structure would be weaker as seen by a 22% reduction in Young’s modulus for resist containing 2% quencher compared to the original resist. 

**Figure 9:**
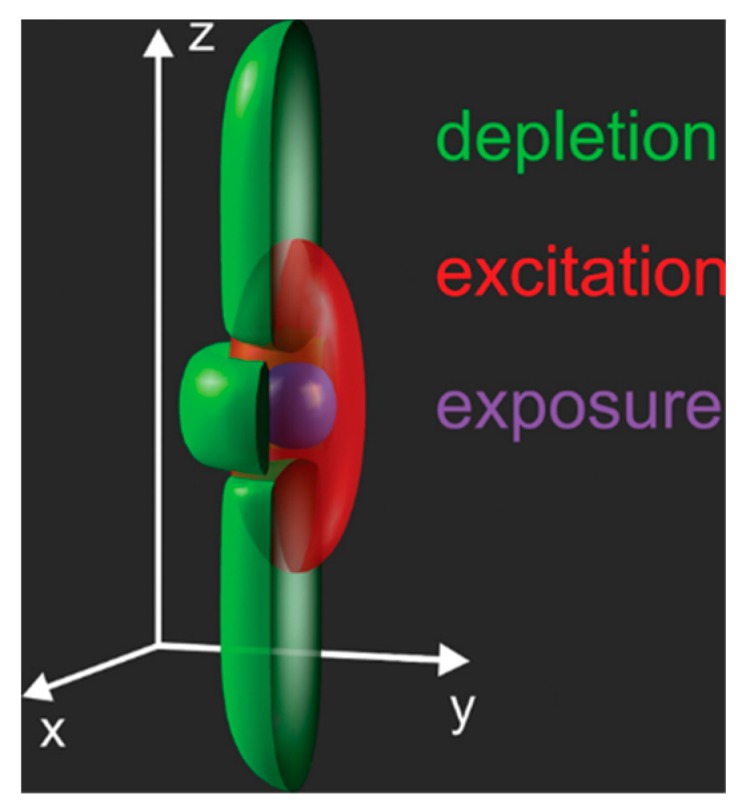
Schematic showing the point spread functions in stimulated emission depletion two-photon lithography [82]. Here the excitation beam is red, depletion beam taking the form of a bottle-beam mode is green, and this yields an effective exposure as shown in purple.

A methodology known as stimulated emission depletion (STED) TPL utilises two lasers of different wavelength to produce overlapping foci. Here the first laser has a wavelength (λ = 780 nm) [85] tuned to the transition within the photoinitiator molecule and hence produces polymerisation. The second laser is passed through a phase mask, yielding a specific geometric profile at focus. Specifically, a Laguerre-Gaussian [10] mode is usually produced, which takes a ring-like profile. This depletion/deactivation beam (λ = 532 nm) inhibits the polymerisation process. By overlapping laser beams at focus (Figure 9), polymerisation is confined to the central region, where the deactivation beam is not present [82,86]. Using this technique, Wollhofan et al. were able to achieve a feature size of approximately 55 nm and lateral resolution of around 120 nm [85]. This methodology requires a bespoke setup that includes an additional depletion laser and photoresists that are sensitive to the depletion laser wavelength.

**Figure 10:**
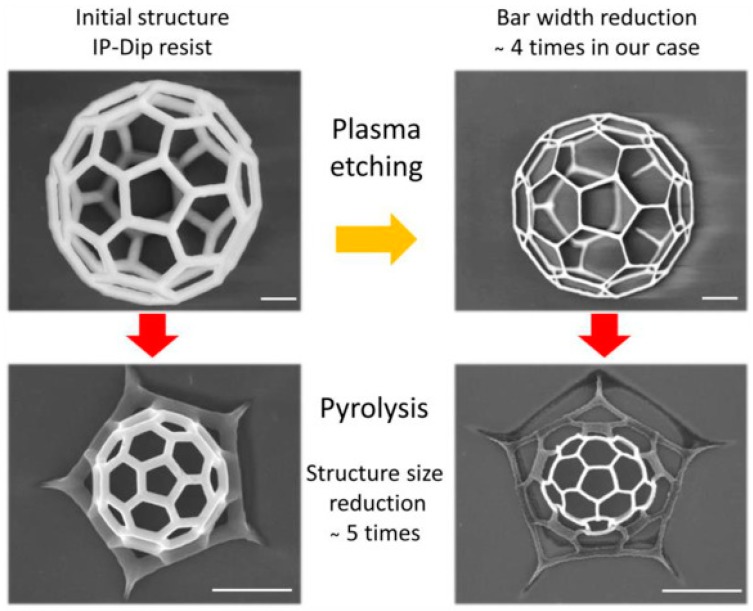
Illustration of reduction in feature size by separate use of oxygen plasma etching, pyrolysis and then a combination of the two techniques [87].

Alternatively, or in addition to the above methods, there are several post-processing techniques that can be applied to TPL printed structures after fabrication to further reduce their feature sizes. One method is pyrolysis, where the structures undergo thermal decomposition to remove the oxygen content from cured polymer. This has the effect of uniformly shrinking the structure in all length directions reducing its overall size by a factor of ~ 5 [87]. However, at regions where the structure is strongly adhered to the substrate deformation will occur during shrinkage. One option for avoiding this is to include a bridging structure between the substrate and nanostructure during the design and fabrication process. A second process that can optionally be used alongside pyrolysis or by itself is plasma etching. For a structure of interconnected wires this will reduce the lateral feature size and can be applied before pyrolysis. Figure 10 demonstrates these techniques applied separately and in combination.

While the above use of pyrolysis is suitable for an approach combined with LOS deposition, an alternative to create solid metallic structures is to dope the photoresist with metallic nanoparticles. The resist is then used with standard TPL to fabricate arbitrary 3D designs with a magnetic material embedded within the structure. Pyrolyzing the doped resist causes the metallic nanoparticles to clump together to form solid wires between 300 and 400 nm wide and 10% and 30% porosity [88]. 

## 5. Outlook

Today, a suite of methodologies is being used to fabricate 3D magnetic nanostructures of various geometry [32]. As is often the case, there is not a single best methodology but rather, upon deciding upon a geometry, one should consider the strengths and weaknesses of individual approaches. For example, it is very clear that electrodeposition into anodised alumina or ion track etched templates is very well suited to producing very pure, cylindrical magnetic nanowires, with very smooth sidewalls and there is little doubt that this powerful methodology will continue to be exploited in order to explore the physics of ultrafast domain walls [34] and eventually spin-Cherenkov physics. Recent work has also shown that for the case of anodised alumina templates, more complex connected nanowire structures can be made [89] suggesting that this methodology may also allow the realization of complex 3D nanowire lattices.

Three-dimensional magnetic nanostructures of arbitrary geometry, which for example may have structures with modulations, curvature or torsion require direct write technologies and here TPL is joined by one other methodology, known as focused electron beam deposition (FEBD). This is a means to directly deposit magnetic structures of arbitrary 3D structure. It is not possible to explore the detailed physics or provide a detailed survey of FEBD literature here, but we refer the reader one of the many excellent reviews upon the subject [90]. A key advantage of FEBD is the resolution that can be obtained [91], potentially surpassing what will be possible with any TPL technique, even after harnessing resolution improvement techniques. Couple this with the fast write times and recent work demonstrating complete flexibility in 3D design [92] and one obtains an incredibly powerful methodology for future 3D nanomagnetism research. There are, however, several drawbacks with this approach, that no doubt with further research will be surmounted. Currently, the choice of magnetic materials that can be deposited is more limited than what could be obtained using evaporation or electrodeposition and this is constrained by availability of suitable precursor gases. Secondly, the growth parameter space is complex [93] and in order to obtain pure deposits such processing parameters need to be carefully controlled. In general, more work is needed to understand the growth process and how best to eliminate carbon-based impurities both within the deposit bulk and upon the surface. Whilst such studies are being done, there is further promise of utilising FEBD to deposit metallic structures and combining this with simple evaporation [94], providing access to systems allowing for domain wall injection from the substrate. 

TPL is also incredibly powerful when combined with techniques to improve the resolution, engineer the point spread function (PSF), and post-process; having the potential to realise ~50 nm isotropic feature size whilst also having significant versatility to be used as a template with electrodeposition or electroless deposition providing access to a wide range of magnetic materials that can be deposited with high degree of purity.

Ultimately, one may envisage multi-scale 3D lithography approaches that utilise TPL to produce 3D magnetic nanostructures whilst other methodologies such as FEBD exploit their improved resolution to produce nanoscale defects upon the surface of the TPL structures, providing access to new spin textures, controlled domain wall/Skyrmion pinning potentials and programmable spin vortex oscillators.
